# Robot Assisted Person-Centered Dementia Care

**DOI:** 10.1093/geroni/igaf122.1541

**Published:** 2025-12-31

**Authors:** Sajay Arthanat, Jing Wang

## Abstract

This symposium explores the development, implementation, and ethical considerations of socially assistive robots (SARs) in dementia care, highlighting their potential to enhance caregiving and support aging in place for individuals with Alzheimer’s disease and related dementias (ADRD). Through an interdisciplinary collaboration between researchers from health science, computer science, and nursing, this work presents a novel SAR equipped with smart sensing capabilities to deliver individualized care protocols tailored to the needs of persons living with dementia (PLWD) and their care partners. First, overview of the project will be shared, highlighting the feasibility, preliminary effectiveness, and user acceptance of SARs in home settings. Second, we will examine the SAR’s technical framework, focusing on the integration of artificial intelligence and perception-action models that enable real-time decision-making in caregiving environments. Third, the symposium will explore the potential of SARs to deliver home-based exercise programs, addressing barriers to physical activity access among older adults, highlighting the use of AI to teach and track exercises and implications for SAR-led rehabilitation interventions. Fourth, we will discuss person-centered SAR care protocols, focusing on care partner feedback and real-world interactions. Findings will highlight design modifications that improve usability, personalization, and engagement for PLWD. Finally, we will address ethical challenges in SAR implementation, emphasizing strategies to balance autonomy with safety, protect privacy, and foster trust in SAR-assisted caregiving. These presentations will provide a comprehensive perspective on how SARs can be effectively designed, implemented, and evaluated to enhance dementia caregiving while maintaining ethical and person-centered care principles.

